# Effect of the Fiber Treatment on the Stiffness of Date Palm Fiber Reinforced PP Composites: Macro and Micromechanical Evaluation of the Young’s Modulus

**DOI:** 10.3390/polym12081693

**Published:** 2020-07-29

**Authors:** Belgacem Chihaoui, Ferran Serra-Parareda, Quim Tarrés, Francesc Xavier Espinach, Sami Boufi, Marc Delgado-Aguilar

**Affiliations:** 1 Faculty of Science, University of Sfax, LMSE, Sfax BP 802-3018, Tunisia; chihaoui.belgacem@gmail.com (B.C.); Sami.Boufi@fss.rnu.tn (S.B.); 2 LEPAMAP Research Group, University of Girona, Maria Aurèlia Capmany, 61, 17003 Girona, Spain; joaquimagusti.tarres@udg.edu; 3 Chair on Sustainable Industrial Processes, University of Girona, Maria Aurèlia Capmany, 61, 17003 Girona, Spain; 4 PRODIS Research Group, Department of Organization, Business, University of Girona, Maria Aurèlia Capmany, 61, 17003 Girona, Spain; francisco.espinach@udg.edu

**Keywords:** date palm fibers, stiffness, composite materials, xylanases, pectinases

## Abstract

The present work aims at determining the potential of date palm wastes to be applied as reinforcement in polypropylene. For this, fibers were separated from the raw biomass via mechanical defibration in Sprout Waldron equipment. Then, three different treatment strategies were adopted on the fibers, being (i) mechanical, (ii) chemical with NaOH, and (iii) enzymatical with xylanases and pectinases. Fibers were characterized in terms of chemical composition, morphology and SEM. Additionally, PP was reinforced with date palm fibers and the composites’ stiffness was evaluated. The analysis was performed from a macro and micro mechanical viewpoint. The incorporation of 40 and 60 wt.% of DPF-E enhanced the Young’s modulus of PP by 205 and 308%, respectively. The potential of enzymatically treated fibers to replace glass fibers in composites was studied, exhibiting similar stiffening abilities at 60 wt.% of date palm fiber (6.48 GPa) and 40% of glass fibers (6.85 GPa). The intrinsic Young’s modulus of the fibers was set at values around 16, 20 and 24 GPa for mechanical, chemical and enzymatic fibers. From the micromechanical analysis, the efficiency of the reinforcement as well as the contribution of the length and orientation to the Young’s modulus of the composite was evaluated.

## 1. Introduction

Polymeric materials have replaced many conventional materials for various applications, mainly due to their ease of manufacture, lower weight, and lower processing costs [1]. However, for some applications, the neat properties that polymeric materials exhibit do not meet the market requirements, inducing the use of reinforcing agents [2,3]. Synthetic fibers (namely carbon, aramid or glass fibers) have been extensively studied and found to provide significant enhancement in terms of mechanical properties. Nonetheless, such reinforcements are known to be abrasive for the processing equipment and even harmful for the human being [4]. Thus, the use of natural fibers has recently attracted attention both at research and manufacturing level, mainly due to their lower cost, lower specific weight and sustainable character compared to the abovementioned synthetic fibers, while providing reasonable mechanical enhancement [1,5,6]. Nevertheless, a certain drawback of natural fiber composites is the incompatibility between the hydrophilic fibers and hydrophobic matrix, which often leads to weak bonding at the interphase. Consequently, the stress is inefficiently transferred from the matrix to the reinforcement [7]. Although this poor compatibility between the phases is not critical to the stiffness of composite materials, the inclusion of a coupling agent to promote the interactions between the fibers and matrix is interesting to improve the strength and deformation capacity in the resulting composite [8,9]. The addition of coupling agents also reduces the water absorption of composites [10]. Hence, the use of coupling agents implies a competitive advantage in comparison to uncoupled composites [11].

Polyolefins are the most widely used polymers in composites for sectors such as the automotive and construction industries, and other consumer products [12]. Among polyolefins, polypropylene (PP) is the most representative [13]. The primary advantages of using PP as a polymer matrix include its favorable strength and stiffness, cost efficiency, chemical and weather resistance and relatively low processing temperature requirements, which are of interest because of the low thermal stability of natural fibers [14]. In PP-based composites, the use of maleic anhydride polypropylene (MAPP) has been effectively used to improve the adhesion between the fiber and matrix [15,16]. On the one hand, MAPP forms chemical bonds with the hydroxyl groups in the fiber surface by means of the maleic groups in MAPP, whereas on the other hand, MAPP can entangle and co-crystallize with the unmodified PP chains [10].

Natural fibers can be obtained from wood species, annual plants and agricultural residues. Among these resources, agricultural residues are considered to be a promising alternative to the use of other fiber sources, thanks to their huge availability and opportunity to add value to a residue. In this group it is possible to find stalks of most cereal crops, rice husks, coconut fibers, and others. In this context, the high population of date palm (*Phoenix dactylifera*) results in large quantities of wastes generated due to the seasonal pruning of the tree. Based on botanical descriptions, 1000 cultivars can be found in Algeria, 400 in Iran, 370 in Iraq, 250 in Tunisia, 244 in Morocco, and 400 in Sudan, among the most important [17]. These wastes are generally burned or landfilled, resulting in serious environmental issues [18,19].

Date palm waste is particularly attractive due to its high abundance and low cost in the industrial and manufacturing sector. In comparison with other plant fibers currently used in industrial sectors, the world production of date palm accounted for 4,200 thousand tons, while sisal, hemp or coir exhibited a production between 100 and 400 thousand tons [20]. On the contrary, out of the abovementioned resources, date palm is the cheapest (0.05 USD/kg), followed by coir (0.3 USD/kg), sisal (0.95 USD/kg) and hemp (1,2 USD/kg) [20]. Furthermore, the relatively high content of cellulose of date palm provides the material with reasonably good mechanical properties [21].

Generally, date palm wastes have been incorporated into polymer composites as powder-like filler, sometimes being sieved to equalize the particle size distribution [22,23,24]. Nonetheless, several techniques can be adopted to extract fibers from the waste material. These techniques can be categorized in three main groups: mechanical, chemical and enzymatic procedures [25,26]. Mechanical treatments aim at fiber extraction via high-yield processes, without severely affecting the chemical composition, and low morphological aspect ratios (length/diameter) are obtained. Alkaline treatments, widely used in the pulp and paper industry, solubilize part of the lignin and carbohydrates present in the lignocellulosic resource structure. This inevitably modifies the fiber chemical and morphological composition, finally affecting the performance of the fibers inside the composite. Disadvantageously, these treatments involve environmental issues related to the low optimization of the material and use of chemical reagents. Alternatively, the use of appropriate enzymes could provide a sustainable solution to these problems [27]. Enzymes modify the structure of the chemical constituents, degrading them and making their removal easier. In this sense, the enzymatic action is characterized by its high selectivity, which can lead to higher optimization of the raw biomass. In addition, employment of chemical reagents is reduced or even avoided [28]. The main enzymes playing a role in the enzymatic extraction of natural fibers are pectinases, xylanases, laccases and cellulases [29]. Pectinases aim at the removal of pectin, xylanases break down the hemicellulose material, cellulase degrades the cellulose polymers and laccase is responsible for the degradation of lignin structure [30]. Recently, the use of enzymes xylanases has increased significantly at the industrial level [31,32]. These enzymes have attracted the attention for biobleaching of pulps, fabric bioprocessing, wastepaper recycling sequences, among others [33]. To improve the accessibility of xylanases to xylans, pectinases can be added for the removal of pectins, which are mainly found at the outer layers of the fiber cell wall between the middle lamella and primary wall. Indeed, previous studies show that the combination of pectinase and xylanase can lead to an effective fiber extraction [34,35].

The development of novel materials might fill demands that cannot be satisfied with the existing materials. Stiffness, strength and dimensional stability are considered the most relevant properties for determining the viability of a material for most of the structural and semi structural applications [36]. For instance, stiffness of composites materials is considered more important than the ultimate strength in products such as door panels, seat backs, and windows, which are currently commercialized products in the field of composites. Stiffness can be easily evaluated by the Young’s modulus parameter. Additionally, the use of micromechanical modelling can be useful to understand the fiber reinforcing mechanisms within a polymer matrix, allowing the prediction of macromechanical properties in similar systems.

In the present work, the use of date palm wastes (DPW) as a potential source of fibers for polypropylene (PP) reinforcement was evaluated. Fibers from DPW were extracted by means of (i) mechanical, (ii) chemical and (iii) enzymatic treatments. To promote the interactions between the matrix and the reinforcement, a coupling agent based on maleic anhydride-modified polypropylene (MAPP) was incorporated into the composites during compounding. In all cases, Young’s modulus was adopted as a reference parameter, both as a macro- and micromechanical property. Overall, the current investigation sheds light on the possibility of valorizing date palm wastes in the field of plastic composites by following different treatment strategies to take advantage of their stiffening potential.

## 2. Materials and Methods

### 2.1. Materials

Composite materials were obtained using polypropylene (PP) as polymer matrix, maleic anhydride polypropylene (MAPP) as coupling agent, and date palm waste (DPW) fibers as reinforcement. Polypropylene (Isplen PP090 G2M) was supplied by Repsol Quimica, S.A. (Tarragona, Spain). According to the supplier, this PP exhibits a density of 0.905 g/cm^3^ and a melt flow index of 35 g/10 min at 210 °C, tested with a weight of 2.16 kg. MAPP was provided by Eastman Chemical Products (San Roque, Spain) under the trade name Epolene G-3015. MAPP exhibits a density of 0.913 g/cm^3^. Date palm waste (DPW) was extracted from different part of the date palm tree and consisted of a mix of leaflets, leaves and rachis, resulting from the annual pruning of the plant in the region of Gabes (Tunisia). Pectinases (Pectinex^®^ XXL) and xylanases (Panzea^®^) were provided by Novoenzymes (Bagsværd, Denmark).

For comparison purposes, composite materials using glass fibers as reinforcement were prepared. Sized E-glass fibers (GF) were produced by Vetrotex (Sant-Gobain, France) and provided by Maben S.L (Banyoles, Spain). All reagents used in this investigation were provided by Sigma Aldrich (Barcelona, Spain) and were used as received.

### 2.2. Methods

**Figure 1:**
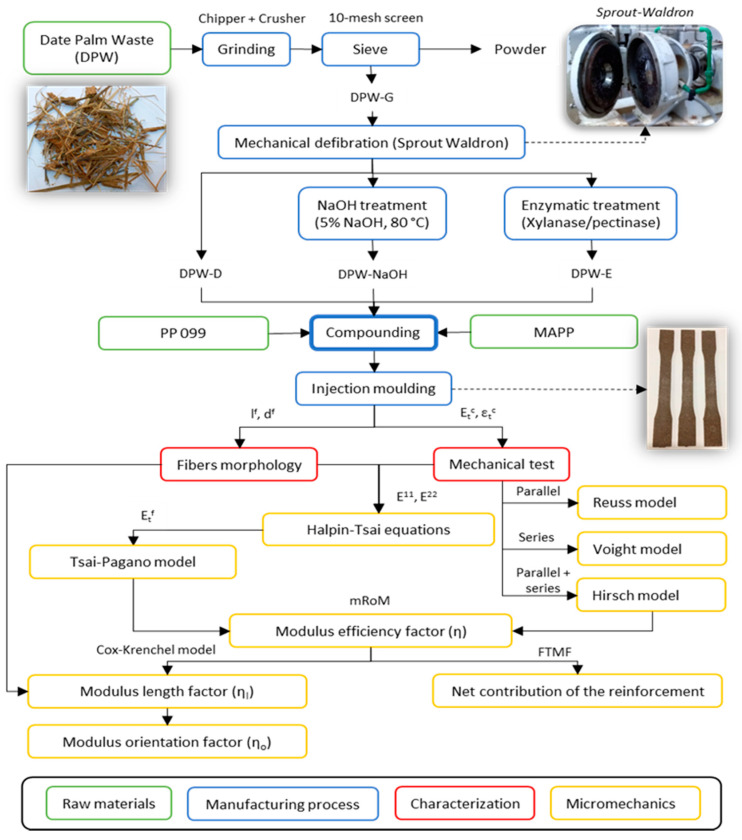
Flowchart of the present investigation.

Figure 1 presents the general workflow of the present study, form the extraction of date palm fibers and production of composite materials, to the final characterization and micromechanical analysis of the materials.

#### 2.2.1. Fiber Extraction from Date Palm Wastes (DPW)

DPW was initially dried at room temperature and then grounded to obtain particles of 10–30 mm length. After this, the chips were mechanically defibrated in a Sprout Waldron equipment (model 105-A, Muncy, PA, USA) to obtain a fibrous like material (DPF-D). Then, the DPF-D was subjected to two different treatments. On the one hand, the defibrated material was submitted to a soft sodium hydroxide (NaOH) treatment catalyzed with anthraquinone (AQ). The reaction was carried out at 80 °C for 2 h, using a 5 wt.% of NaOH and 0.1 wt.% of AQ with respect to the fiber content. The liquid-to-solid ratio was set at 7:3. Then, the resulting fibers were washed with distilled water to remove any excess of non-reacted reagents and impurities. The resulting suspension was labeled as DPF-NaOH. On the other hand, DPF-D was treated with enzymes pectinase and xylanase. Initially, the enzymes were dissolved in their respective buffer solutions. Xylanase was dissolved in phosphate buffer at pH 7, and pectinase in acetate buffer at pH 4.8, for a total volume of 1 L each. 50 g of DPF-D were then mixed with 1L of the enzyme solution at rates of 100 U/g for xylanase and 3000 U/g for pectinase, with respect to the fiber content. The suspension was kept under constant stirring for 24 h at a temperature of 50 °C. The enzymatically treated fibers received the name DPF-E.

Finally, the treated fibers were oven dried until constant weight for further use and characterization.

#### 2.2.2. Chemical Characterization of the Fibers

The chemical constituents of the fibers were determined from the measurement of the ethanol soluble extractives (TAPPI T204 cm-07), ashes (ISO 2144:2019) and Klason lignin (ISO 2144:2019 standard). The holocellulose was measured by difference. The kappa number was determined following TAPPI T236 om-06.

#### 2.2.3. Composite Compounding

Composites were prepared at fiber contents of 40 and 60 wt.%. For comparison purposes, composites containing 10, 20, 30 and 40 wt.% of E-glass fiber were also prepared. The compounding process was carried out in a Brabender^®^ plastograph internal mixer (Brabender GmbH & Co KG, Duisburg, Germany). Temperature was set at 180 °C regardless the type of reinforcement, while rotational speed was set at 20 rpm in the case of glass fiber and at 80 rpm for those fibers from DPW. All the process was computer-controlled by means of WINMIX software. First, PP and MAPP were added to the mixing chamber until no torque variation was observed. Then, the reinforcement was added at the abovementioned ratios and kept under mixing until constant torque. The obtained materials were then cooled down at room temperature and ground in a knives mill Retsch SM 100 (Retsch Iberia, Llanera, Spain). The obtained granules were stored at 80 °C in an oven to prevent moisture absorption prior to injection molding.

#### 2.2.4. Composite Processing

Dog-bone standard specimens were produced in an Aurburg 220 M 350-90U injection-molding machine (Aurburg, Loßburg, Germany) complying with ASTM D3641. The injection molding process was performed at temperatures of 175, 180, 185, 185 and 190 °C. The use of higher temperatures was avoided to reduce the thermal degradation of the fibers. Besides, the pressure was ranged between 300 and 600 bar depending on the fiber content. Ten specimens from each composite material were produced and stored in a climatic chamber at 23 °C and 50% of relative humidity for 48 h, as required by ASTM D618 standard.

#### 2.2.5. Tensile Test

Tensile tests were carried out in an Instron TM 1122 universal testing machine (Metrotec, Lezo, Spain) fitted with a 5 kN load cell and according to ASTM D618. A gap between claws was set at 115 mm with a crosshead testing velocity of 2 mm/min. For the Young’s modulus determination, an MFA 2 extensometer (Walter + Bai AG, Löhningen, Switzerland) was used for a more precise deformation measurement. In parallel, the tensile strength and deformation at break of the specimens was measured. The tensile strength was needed for the adjusting the amount of the coupling agent in the composites, whereas the deformation at break was measured to evaluate how the variations of the Young’s modulus affected the deformation capacity of the composites.

#### 2.2.6. Fiber Extraction from the Composites and Characterization

Injected composites were submitted to Soxhlet extraction using decahydronaphthalene as solvent to dissolve the PP matrix. The extraction was performed in 48 h. The solid residue and the fibers were vigorously washed with acetone to remove any impurities. Fibers were then suspended in water to prevent their agglomeration.

The extracted fibers were characterized from a morphological point of view by means of a MorFi Compact Analyzer by Techpap SAS (Grenoble, France). The analyzer is equipped with a CCD video camera and controlled by MorFi v9.2 software. The equipment is able to measure 30,000 fibers per batch (1 L per batch at 25 mg/L of concentration). The equipment provides the mean fiber length and diameter, among others.

#### 2.2.7. Micromechanical Analysis of the Young’s Modulus

Despite all existing information on natural fiber composites, its complex behavior under load, different to traditional materials, and high degree of anisotropy requires a study in depth to take advantage of its potential. In this context, micromechanical models provide information on the expected properties of composite materials.

The intrinsic Young’s modulus of the fibers was computed following two different methodologies: (i) Hirsch model and (ii) Tsai–Pagano model using Halpin–Tsai equations. Comparatively, the Hirsch model uses only experimental data from the mechanical test, whereas the incorporation of Halpin–Tsai equations takes into consideration the morphological characteristics of the fibers [37].

Hirsch model defines a linear combination between the parallel, Reuss, and the series, Voight, models [38]. The models are computed as follows.

Parallel model.

(1) $$E_{t}^{c}=E_{t}^{f}\cdot V^{f}+E_{t}^{m}\left(1-V^{f}\right)$$

Series model.

(2) $$E_{t}^{c}=\frac{E_{t}^{f}\cdot E_{t}^{m}}{E_{t}^{m}\cdot V^{f}+E_{t}^{f}\cdot \left(1-V^{f}\right)}$$

Hirsch model.

(3) $$E_{t}^{c}=\beta \cdot \left(E_{t}^{f}\cdot V^{f}+E_{t}^{m}\left(1-V^{f}\right)\right)+\left(1-\beta \right)\frac{E_{t}^{f}\cdot E_{t}^{m}}{E_{t}^{m}\cdot V^{f}+E_{t}^{f}\cdot \left(1-V^{f}\right)}$$

**Figure 2:**
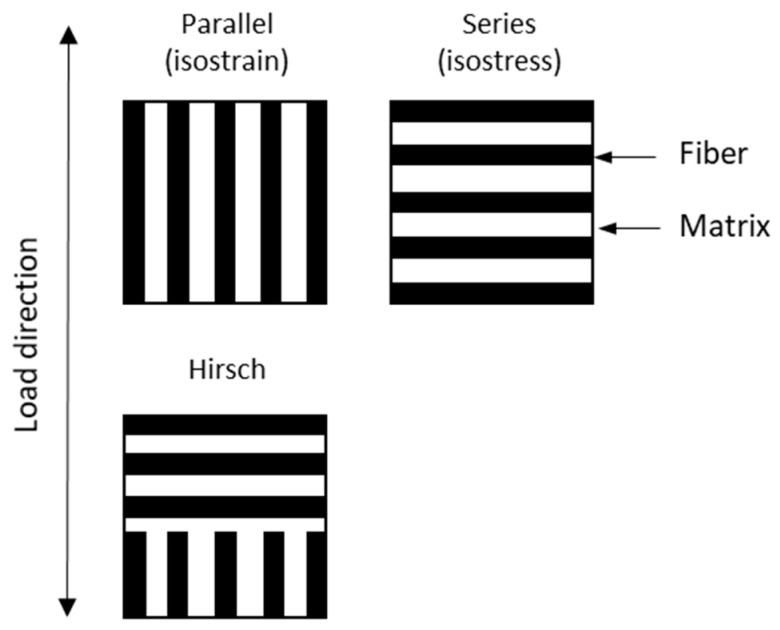
Parallel, Series and Hirsch model representation in composite materials.

$E_{t}^{c}$ and $E_{t}^{m}$ are the Young’s modulus of the composite and matrix, respectively, obtained from the mechanical test, and $E_{t}^{f}$ is the intrinsic Young’s modulus of the fiber. The fiber volume fraction inside the composite is represented by $V^{f}$. β defines the stress transfer between fiber and matrix. The value is strongly influenced by the orientation, length and concentration effect at the end of the fibers [39]. A value of β = 0.4 has been found to be accurate with experimental behavior in semi-aligned fiber composites processed by means of injection molding [40]. For the reader’s convenience, the serials, parallel and Hirsch model are represented in Figure 2.

The Tsai and Pagano model [41] and the Halpin and Tsai [42] equations were also used to calculate the intrinsic Young’s modulus of fibers. Tsai–Pagano’s model is described as.

(4) $$E_{t}^{C}=\frac{3}{8}E^{11}+\frac{5}{8}E^{22}$$

Being $E^{11}$ and $E^{22}$ the longitudinal and transverse modulus of the composite material, respectively. These factors are determined by the Halpin–Tsai equations (Equations (5) and (6)).

(5) E11=1+2(lFdF)·ηlVF1−ηlVFEtm

(6) $$E^{22}=\frac{1+2\cdot \eta_{t}V^{F}}{1-\eta_{t}V^{F}}E_{t}^{m}$$

$\eta_{l}$ (Equation (7)) and $\eta_{t}$ (Equation (8)) are parameters calculated as.

(7) ηl=(EtFEtm)−1(EtFEtm)+2(lFdF)

(8) ηt=(EtFEtm)−1(EtFEtm)+2

Both Hirsch and Tsai–Pagano models define a combination of the Young’s modulus in composites when the load is exerted longitudinally and transversally to the fiber direction. Thereby, a comparison can be stablished between the parallel model and $E^{11}$, and the series model and $E^{22}$.

To correct the contribution of natural fibers to the stiffness of the composite, the modulus efficiency factor (η) was determined thanks to the modified Rule of Mixtures (mRoM) as described in Equation (9) [16,43].

(9) $$E_{t}^{c}=\eta \cdot E_{t}^{f}\cdot V^{f}+E_{t}^{m}\cdot \left(1-V^{f}\right)$$

The modulus efficiency factor (η) can be expressed as the product between the modulus length factor $\left(\eta_{l}\right)$ and modulus orientation factor $\left(\eta_{o}\right)$. Hence, it is possible to evaluate the contribution of the orientation and length to the effectiveness of the reinforcement. The modulus length factor can be calculated following the Cox-Krenchel model (Equation (11)). Then, the orientation factor is isolated from the relationship between the factors.

(10) ηl=1−tanh(β·lf2)(β·lf2)

where β is the coefficient of stress concentration rate at the end of the fibers, defined as (Equation (11)).

(11) β=1rEtmEtf·(1−ν)·Lnπ4·Vf

r and $l^{f}$ are the mean fiber radius and diameter, respectively. The Poisson’s ratio of the matrix is expressed by ν, which in the case of PP is 0.36 [44].

The neat contribution of the fibers to the Young’s modulus of the materials can be estimated by the Fiber Tensile Modulus Factor (FTMF), in accordance with Thomason methodology [45]. This factor is obtained by representing $E_{t}^{c}-E_{t}^{m}\cdot \left(1-V^{f}\right)$ as a function of the fiber volume fraction ($V^{f}$) (Equation (12)). The slope of the line $\left(\eta \cdot E_{t}^{f}\right)$ will determine the contribution of the fibers to the Young’s modulus of the composites. The FTMF has been effectively used in other works to evaluate the stiffening capabilities of different fibers in polymeric matrices [46,47].

(12) $$FTMF=\eta \cdot E_{t}^{f}=\frac{E_{t}^{c}-E_{t}^{m}\cdot \left(1-V^{f}\right)}{V^{f}}$$

## 3. Results

### 3.1. Effect of the Coupling Agent

**Figure 3:**
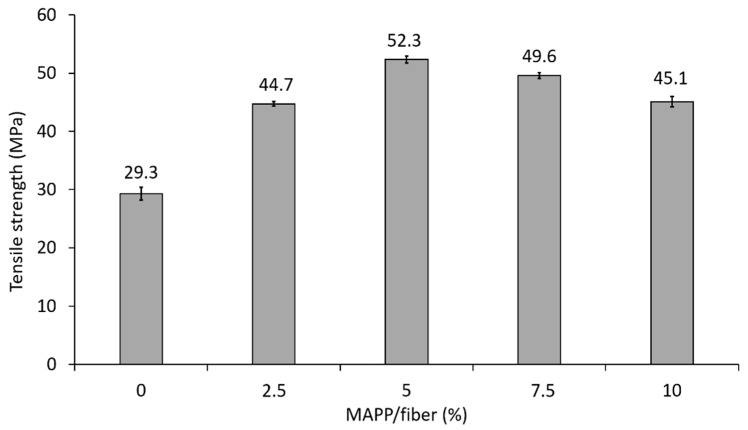
Tensile strength of composites reinforced at 40 wt.% of DPF-E and different MAPP contents.

As mentioned in the introduction, the Young’s modulus of composites is not significantly affected by the strength at the interphase; thus, the incorporation of MAPP would not be necessary if the purpose was the enhancement of composites’ stiffness. Nevertheless, the use of coupling agents supposes a competitive advantage over uncoupled composites in view of other mechanical parameters such as strength and deformation. Hence, the amount of coupling agent adjusted to achieve a good interfacial adhesion. The tensile strength property was adopted as reference, as it is known that the fiber–matrix interface has an impact in the tensile strength of composites [8]. The influence of MAPP (0, 2.5, 5, 7.5 and 10 wt.% MAPP with respect to fiber content) was evaluated for PP composites with a 40 wt.% of DPF-E. When the amount of MAPP set, the same MAPP percentage was applied to the rest of the composites. The tensile strength of the resulting composites is shown in Figure 3.

The composite material without MAPP exhibited a similar tensile strength to polypropylene (27.6 MPa), which evidences scarce fiber–matrix compatibility. The higher values of the tensile strength were measured at a 5 wt.% MAPP percentage, which is indicative of an optimal interfacial union. The reduction of the tensile strength at lower MAPP contents is attributed to the lack of adhesion between the phases, whereas at higher percentages, the decrease of the strength is ascribed to self-entangling and slippage of MAPP chains [15].

**Figure 4:**
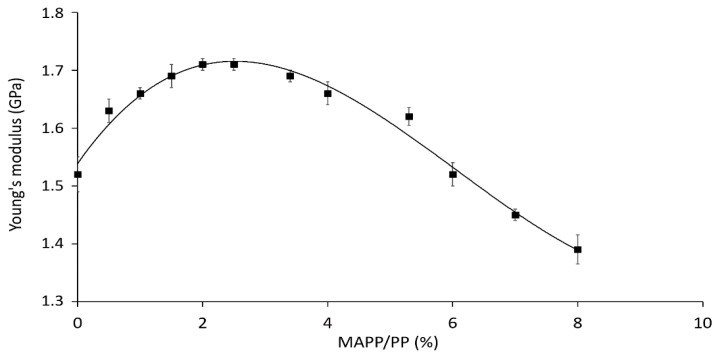
Effect of MAPP on the Young’s modulus of PP.

In principle, the incorporation of the coupling agent to the composites could affect the stiffness of the neat matrix. Thereafter, in order to have a realistic approach on the effect of fiber content on composites’ stiffness and to validate the use of micromechanical models, the Young’s modulus of the matrix was corrected. To study the effect of MAPP on the Young’s modulus of the matrix, PP/MAPP blends at different compositions were prepared, and their Young’s modulus measured. Results are presented in Figure 4.

The addition of MAPP had a slight effect on the stiffness of the matrix, as observed in Figure 4. The Young’s modulus of polypropylene without coupling agent was 1.50 GPa. The addition of MAPP increased the stiffness of the blends up to 1.71 GPa at 2.5% MAPP/PP, then the property decreased again to 1.39 GPa at 8% MAPP/PP. This could indicate that the larger adhesion between MAPP and PP is achieved at a 2.5 wt.% of MAPP/PP, whereas at higher MAPP contents, the presence of a less rigid phase reduces the stiffness of the blend.

In the current investigation, an optimal content of MAPP to enhance the fiber-matrix adhesion was set at a 5 wt.% with respect to fiber content. Considering that the formulated composites were reinforced with a 40 and 60 wt.% of DPF, the corresponding percentages of MAPP/PP at these compositions would be 3.3 and 7.5 wt.%, respectively. At these points, the Young’s modulus of the matrix accounted for 1.70 and 1.41 GPa, respectively. Subsequently, these values were used as the matrix stiffness during the macro and micromechanical analysis.

### 3.2. Macro-Mechanics of the Composites

Composite materials based on polypropylene (PP) and date palm fibers (DPF) were prepared at reinforcement contents of 40 and 60 wt.%. Table 1 shows the obtained values for date palm fibers (DPF) and glass fiber (GF) reinforced polypropylene composites. In the table it is possible to see the reinforcement volume fraction ($V^{f}$), Young’s modulus of the composite $\left(E_{t}^{c}\right)$, deformation at break $\left(\varepsilon_{t}^{c}\right)$ and bulk density ($\rho^{c}$) of the obtained materials.

The incorporation of both natural fibers and glass fibers have been reported to improve the Young’s modulus of materials [48,49]. Thus, as expected, the incorporation of any of the obtained date palm fibers (DPF) enhanced this property. However, the improvement in Young’s modulus was not the same for all the types of DPF, achieving the maximum values with the DPF-E, and the lowest when DPF-D were incorporated into the PP matrix. As will later be discussed, both alkali and enzymatic treatment decreased the lignin content of the fibers, as well as part of the hemicellulose. Both constituents, lignin and hemicellulose, constitute the major part of the amorphous regions of the lignocellulosic biomass, which may present lower stiffness than alpha cellulose. As these constituents are removed from the fibers, the intrinsic Young’s modulus is expected to increase. Statistical ANOVA analysis at 95% confidence rate revealed significant differences in the Young’s modulus of composites depending on the treatment applied. The incorporation of 40 and 60 wt.% of DPF-E enhanced the Young’s modulus of PP in 205 and 332%, respectively. Comparatively, only 20 wt.% of GF was required to obtain a Young’s modulus of the same magnitude as in the case of 40 wt.% reinforced DPF composites, regardless of the treatment, and 40 wt.% to reach similar values to those 60 wt.%-reinforced DPF composites. Furthermore, DPF showed higher stiffening abilities than other natural fibers obtained from wood [50] and agricultural residues [51], though, slightly below the values obtained for annual plant filaments such as hemp strands [43].

**Figure 5:**
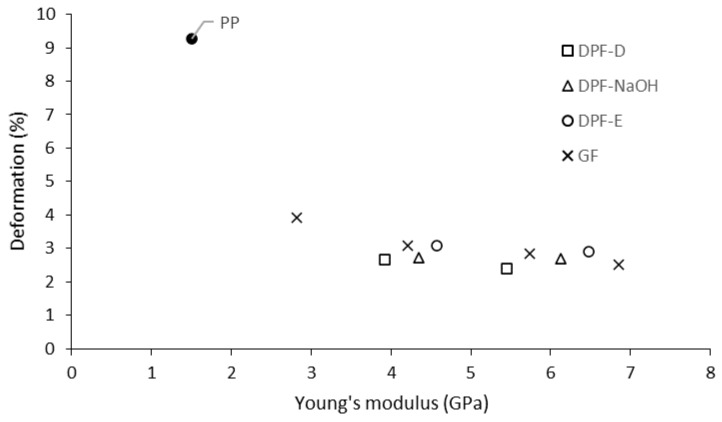
Evolution of the deformation with the Young’s modulus of the composites.

As expected, the greater stiffness of the composites resulted in a reduction on the deformation capacity (Figure 5).

**Figure 6:**
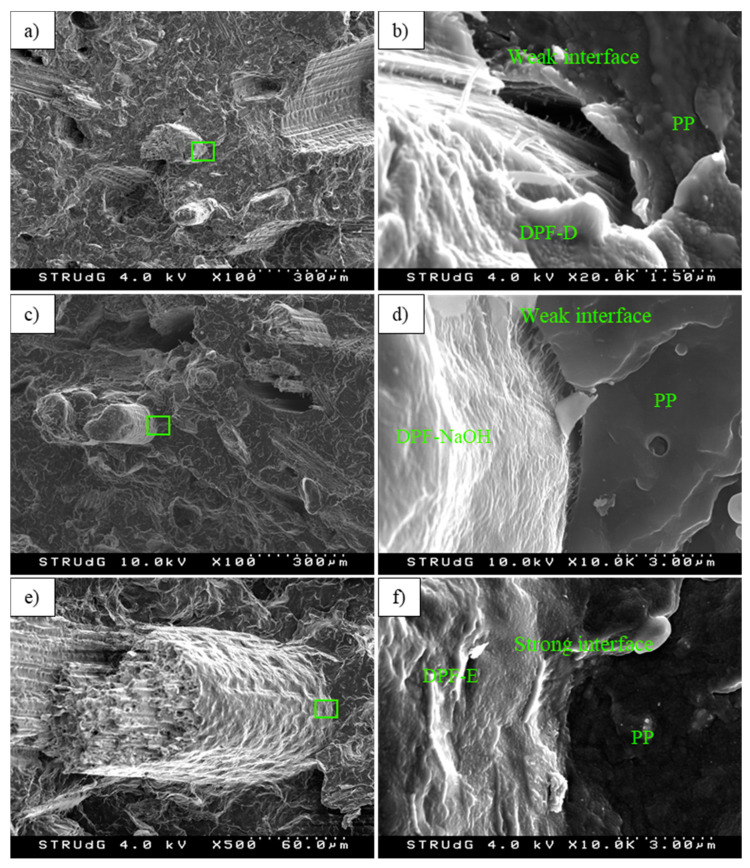
SEM images at the cross-sectional area of the composite specimens, at different magnifications. (**a**,**b**) PP + DPF-D; (**b**,**c**) PP + DPF-NaOH; (**e**,**f**) PP + DPF-E.

DPF-E reinforced composites showed the highest values of deformation at break. The deformation at break in natural fiber composites is affected by the reinforcement volume fraction, the dispersion within the matrix and the interaction between the reinforcement and the matrix [52]. Thereby, low fiber–matrix interactions could finally drive to stress concentration effect in the shape of material discontinuities, such as voids, promoting the fracture. In this context, the coupling agent plays a major role on improving the stress transfer ability between phases. In view of the deformation capabilities of the composites, one can expect a higher compatibility of DPF-E compared to DPF-D and DPF-NaOH with the polymeric phase. To further assess the quality at the interface between the fiber and the matrix, already-tested composites were observed by means of scanning electron microscopy (SEM) at the cross-sectional area (Figure 6).

In Figure 6a,c, several holes in the PP matrix can be observed, presumably due to fiber slippage during mechanical testing. This brings to the light a weak interface between PP and DPF-D and DPF-NaOH. However, observing the DPF-NaOH-reinforced composites at high magnification, some interactions can be observed between fibers and PP. This could explain the higher Young’s modulus and elongation at break of DPF-NaOH composites compared to those reinforced with DPF-D. DPF-D exhibited the highest surface fibrillation, mainly due to the treatment conditions. This higher fibrillation may contribute to the mechanical anchorage. However, no significant improvement was observed in Young’s modulus, as both DPF-NaOH and DPF-E exhibited highest values. Considering Figure 6e, it becomes apparent that DPF-E exhibited the best interface compared to the rest of DPF. Apparently, no voids could be observed in the PP matrix, and fibers seem to be considerably attached to the matrix (Figure 6f). The better interactions of one type of DPF compared to another may be explained by different effects, being the mechanical anchorage and the surface chemical composition those playing a key role in fiber bonding. Indeed, this interfacial enhancement can be explained by the modifications in the fiber’s chemical composition. In this regard, the increment of hydroxyl groups given by the holocellulose content leads to an increment in the bonding ability by means of maleic groups present in MAPP [53]. Indeed, the shear strength transferred from the matrix to the fiber bundles starts the loading of such phases. Fiber bundles are composed of single fibers and the interface between such fibers, mainly lignin and hemicellulose. Thus, the same mechanisms of load transferring observed in the composite between the matrix and the reinforcement, are repeated inside the reinforcement at other scale [54]. The short deformations used to measure the Young’s modulus prevent the failure of the matrix–fiber interface and also of the fiber–fiber interface. The chemical composition of the different DPF compared to DPW is collected in Table 2.

The highest content of lignin, extractives and ashes were measured in mechanically defibrated fibers, recording lower amounts of holocellulose. This was expected, since neither chemical nor enzymatic treatment was applied to modify the composition of the fibers. Furthermore, the extractives, ashes and lignin contents indicated that the raw material contained high quantities of leaflets, with minor parts of leaves and rachis.

For NaOH-treated fibers, a decrease on the ashes and extractives contents was measured. The content of lignin also decreased, but in less extent. As a result, the holocellulose content incremented. A change in the morphology of the fibers is also expected because of the alkaline treatment. In this sense, lignin, along with some ashes and extractives, are mainly found in the primary wall and middle lamella, acting as a cementing material between the fibers. Hence, the weakening of these compounds by the alkaline effect leads to a better separation of the fibers [53]. Such effects on morphology will be later observed.

During the enzymatic treatment, a major reduction of the lignin, extractives and ashes content was observed. Pectinase and xylanase aim at the structural modification of pectins (hemicelluloses) and xylans (extractives), respectively. Hence, the effect of the enzymes on the reduction of hemicellulose and extractives was expected during the enzymatic treatment. However, the degradation of these compounds may inevitably lead to the removal of other constituents contained in the amorphous regions of the fiber cell wall, mainly lignin and inorganic matter. Indeed, lignin reduction accounted for a 25% approximately, which is a significant reduction compared to the alkali treatment.

Between alkali and enzymatic treatment, the action of enzymes can be considered more effective and efficient due to its high selectivity. This is supported by the fact that enzymes cleave indistinctively at the end or in the interior of the carbohydrate’s chains, whereas alkaline treatments are more likely to attack at the end of the chains, reducing the effectiveness of alkaline treatments [56,57]. Overall, the highest content of carbohydrates (cellulose and hemicellulose) was found in DPF-E, which explains the improvement of the fiber-matrix interfacial adhesion.

From Table 1, data from GF reinforced polypropylene is exhibited. PP was reinforced with glass fibers at 10, 20, 30 and 40 wt.%. At GF contents over 40 wt.% the fibers tended to aggregate, reducing the stress transfer efficiency from the matrix to the reinforcement. Moreover, the use of low amounts of GF is usually recommended to prevent equipment attrition [7,58]. For a similar Young’s modulus both in the case of DPF and GF-reinforced composites, neither density nor elongation at break differed much from each other. This is of special interest, since both parameters are of major importance when envisioning the application of composite materials [36]. In addition, the incorporation of higher amounts of reinforcement reduces the use of PP, which may contribute to environmental and economic advantages.

### 3.3. Contribution of the Fibers to the Stiffness of Composites

**Figure 7:**
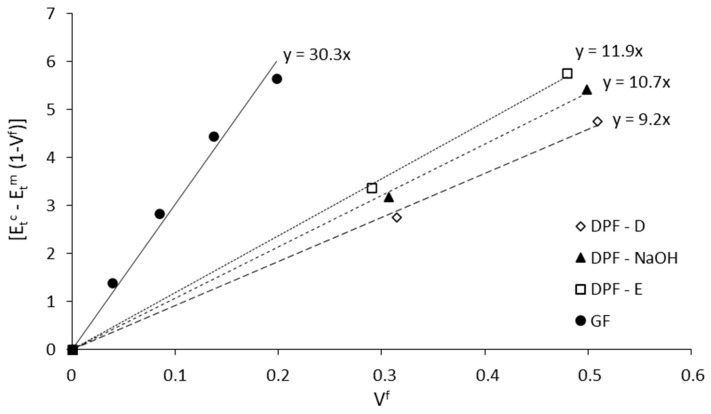
Net contribution of the reinforcements to the stiffness of the composites.

Fiber tensile modulus factor, known as FTMF, is a broadly used method for evaluating the contribution of the reinforcement to the stiffness in a fiber reinforced composite material. This value is considered useful since it serves as comparison with other types of reinforcement and polymeric matrices. This parameter is the slope of the linear regression resulting from plotting the net contribution of the fibers to the stiffness of the composite against the volume fraction of fiber (Figure 7).

The obtained FTMF values were 32.3, 11.9, 10.7 and 9.2 for GF, DPF-E, DPF-NaOH and DPF-D, respectively. The values found for the different DPF were of the same magnitude than those reported in the bibliography for wood fibers, and considerably higher than other fibers from agricultural residues [50,59,60,61].

The net contribution of DPF-E at 60 wt.% was the same as glass fibers at 40 wt.%, thus exhibiting the huge stiffening potential of the natural waste. The net contribution to the stiffness of the materials was slightly lower in alkali treated fibers, whereas in mechanical fibers (DPF-D), a similar contribution was attained at a 60 wt.% of DPF-D and 30 wt.% of glass fibers.

### 3.4. Micro-Mechanical Analysis

The prediction of the fibers’ intrinsic Young’s modulus can be addressed by means of (i) the Hirsch model or (ii) the Tsai–Pagano model using the Halpin–Tsai equations (TP/HT). While the Hirsch model uses only experimental data from the mechanical test, the Tsai–Pagano model along with Halpin–Tsai equations also attains the morphological characteristics of the reinforcing fibers. Accordingly, fibers were recovered from the composite materials via Soxhlet extraction using decahydronaphthalene to dissolve the matrix. Then, the morphology of the reinforcement was evaluated.

The main morphological characteristics of the extracted fibers can be observed in Table 3, as well as the computed intrinsic Young’s modulus of the fibers through the abovementioned models.

As it is possible to see, the different processing methodologies strongly affected the fiber morphology. High diameters around 29.8 µm were attained in DPF-D, resulting in low aspect ratios (length/diameter). This was in part expected, since fibers rupture during purely mechanical processes can occur indistinctively at the different layers of the fiber cell wall. Thereby, the quality of fiber separation is poor, which contributes to low aspect ratios. The diameter decreased to values around 27.5 µm in DPF-NaOH while the length was not significantly affected, thus, the aspect ratio increased in comparison to DPF-D. Additionally, the diameter and length were pronouncedly reduced with the enzymatic treatment (DPF-E) as a result of the chemical constituents breakdown by the enzyme activity. During the compounding process, the fibers’ length was reduced by about 15% from 40 to 60 wt.% reinforcement, regardless of the treatment, whereas the diameter did not appreciably change. Such effect can be attributed to the deterioration of the fibers as a result of the shearing forces created during the mixing of the phases. This deterioration is increased at larger fiber contents, since the viscosity of the mixture increments, and so do the shearing forces [62].

**Figure 8:**
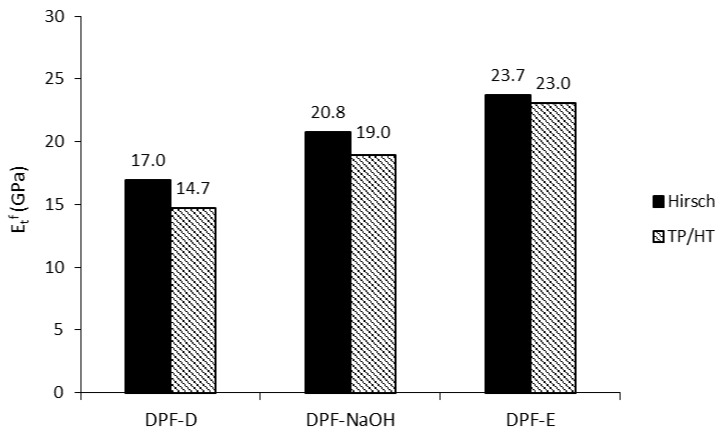
Intrinsic Young’s modulus of date palm fibers computed via Hirsch and TP/HT.

The prediction of the intrinsic Young’s modulus of the fibers returned similar values with the Hirsch and Tsai–Pagano models. The good agreement between methodologies suggests the usefulness and effectiveness of Hirsch model for calculating the intrinsic Young’s modulus with no requirement of morphological data. The similarities between models are better observed in Figure 8, where the mean values of the intrinsic Young’s modulus (40 and 60 wt.% reinforcement) of DPF are represented.

DPF-E exhibited the highest intrinsic Young’s modulus, with values in the range of 23–24 GPa, followed by DPF-NaOH and DPF-D. The mean intrinsic Young’s modulus of DPF-E was higher than the ones found in other works dealing with hardwood and softwood fibers (18–23 GPa) [36,50]. DPF-E also exhibited a higher intrinsic Young’s modulus than other fibers extracted from agricultural residues [26,63].

The effect of the treatment on the intrinsic Young’s modulus of the fibers can be explained by the chemical composition thereof. Indeed, the influence of the lignin content on the intrinsic Young’s modulus of the fibers has been previously discussed in the literature. Neagu et al. [36] reported that the stiffest fibers were likely to be in a kappa range between 30 and 50. In a recent study of the research group [37] dealing with the use of an agricultural residue the range was limited from a 40 to 50 kappa number [37]. Accordingly, one could select the appropriate treatment and operating conditions to obtain fibers in the kappa range of 40 to 50 and finally achieve a more competitive material.

**Figure 9:**
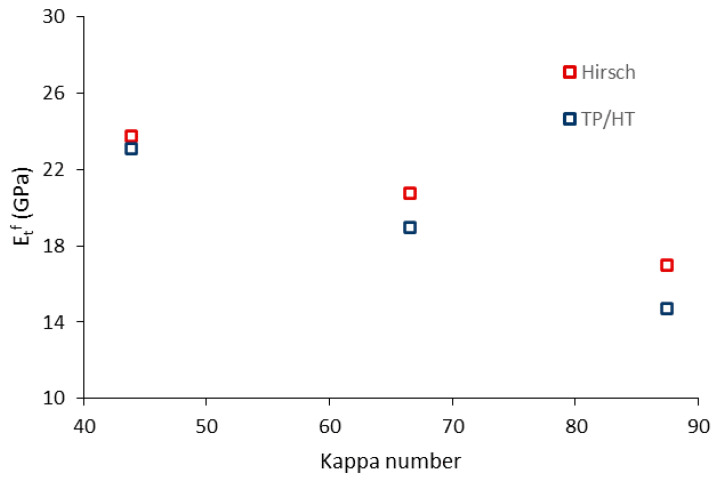
Evolution of the intrinsic Young’s modulus of the fibers with the kappa number.

The fact that a favorable content of lignin is in the kappa range of 40 to 50 is ascribed to the characteristics of the constituents. Natural fibers can be considered as a composite material being cellulose the load-bearing entity and lignin the matrix. Thereby, natural fibers with high lignin and low cellulose contents (Kappa > 50) will possess a lower rigidity due to its high amorphousness. Nonetheless, favorable amounts of lignin are beneficial to improve the stress-transfer capacity between fibers. If such component is partially or completely removed (Kappa < 40), the stress-transfer capacity between fibers drops considerably, and damage may develop even at low strains. Figure 9 presents the evolution of the intrinsic Young’s modulus with the Kappa number.

The stiffest fibers were found at a kappa number of approximately 43, corresponding to DPF-E. Then, it can be stated that the chemical composition in DPF-E favors the creation of a strong interfacial bonding and provides high intrinsic stiffness to the fibers.

The mRoM was applied to obtain the modulus efficiency factor. The factor encloses the effect of the fiber length and orientation distributions. Thereby, the influence of these factors on the efficiency of the reinforcements was computed via the modulus length and orientation factors. The calculus was made using the intrinsic Young’s modulus of the fibers obtained via Hirsch and TP/HT models (Table 4).

The efficiency factor was found to be within the range for short semi-aligned fibers in composites, which in polyolefins is around 0.52 [44,50]. The factor remained almost invariable using the Hirsch modulus, though, larger discrepancies between different types of fibers were recorded using Tsai–Pagano modulus. The Cox and Krenchel model was applied to compute the values of the modulus length factor, obtaining values close to 1. This factor did not vary significantly among the different types of fibers and the different reinforcement contents. Besides, the high values obtained for the length factor suggested the importance of fiber morphology on the composites Young’s modulus.

Finally, it was possible to calculate the value of the modulus orientation factor, expressed as the ratio between the modulus efficiency and length factor. It has been reported that the orientation factor is 1 for completely aligned fibers, 3/8 for planar random configuration and 1/5 for three-dimensional random orientation [64]. In the present case, values around 0.6 were obtained, showing a certain degree of fiber alignment inside the composite. The factor decreased slightly in DPF-D and DPF-NaOH at the time the reinforcement content increased from 40 to 60 wt.%, suggesting difficulties in its dispersion. However, the orientation factor in DPF-E remained unchanged within the different fiber loadings, highlighting the potential of the enzymatic treatment in providing a good interfacial adhesion and dispersion of the reinforcement.

### 3.5. Evaluation of the Longitudinal and Transverse Modulus of the Composites

**Figure 10:**
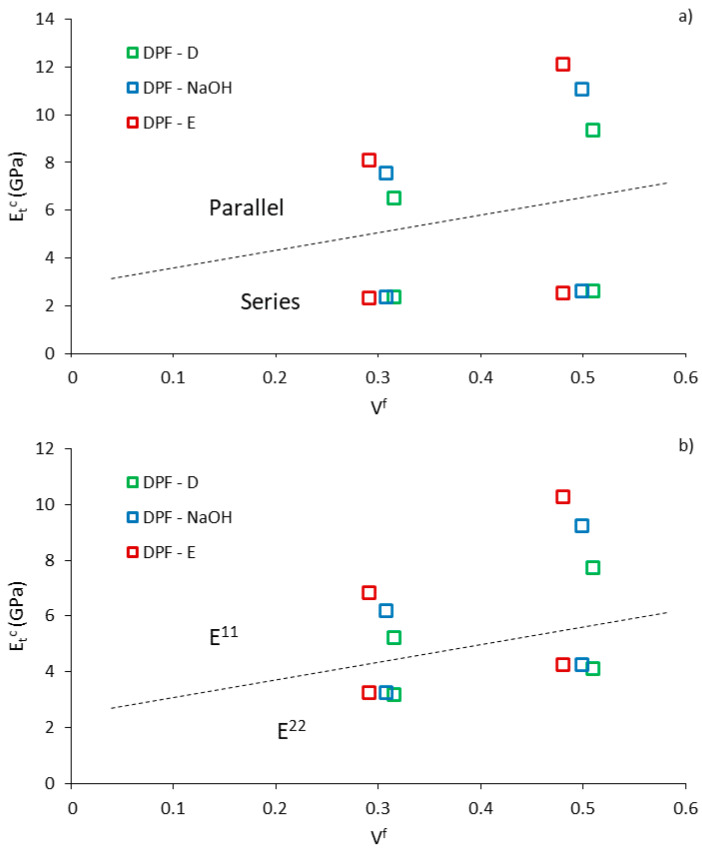
Evolution of the longitudinal and transverse modulus of the composites. (**a**) Parallel and series (Hirsch), (**b**) E11 and E22 (TP/HT).

Both Tsai–Pagano and Hirsch models make a combination of the composite’s longitudinal modulus and transverse modulus. The models consider the existing anisotropy in natural fiber composites, represented by the longitudinal (E^11^) and transverse (E^22^) modulus in the case of Tsai–Pagano, and by the parallel and series model in Hirsch model. This anisotropy in composites is attributed to the anisotropy of the fibers itself [65]. Hence, a good understanding of the material’s anisotropy when considering its potential application is crucial, as the capacity of the composite to withstand the load can vary depending on fiber orientation. Unfortunately, many studies fail in their attempt to model the properties of natural fibers composites due to the supposition of the isotropic properties of fibers [66,67]. Therefore, by using the computed Hirsch and Tsai–Pagano intrinsic Young’s modulus of the fibers, the modulus of the composites in the longitudinal and transverse direction of the fibers can be obtained. The values are presented in Figure 10.

From Figure 10, the modulus of the composite in the longitudinal and transverse direction of the fiber are represented above and below the dashed line, respectively. The stiffness in the transverse direction was considerably lower than in the longitudinal direction, thus, attributing a certain degree of anisotropy. The transverse modulus was not significantly affected by the fiber treatment, though, the differences were pronounced in the longitudinal direction. Such anisotropy can be considered as an advantage since composites are usually employed for structural and semi-structural applications where the stress situation is unidirectional. Furthermore, similarities were found between the parallel model and E^11^, and the series model and E^22^.

## 4. Conclusions

Date palm fibers were extracted from date palm wastes following different mechanical, chemical and enzymatic treatments. Composite materials were prepared by loading polypropylene with a 40 and 60 wt.% of these date palm fibers. The Young’s modulus of the composites was investigated as an important property determining the suitability of the material for various applications. At 40 and 60 wt.% reinforcement, remarkable increments in the Young’s modulus of about 205% and 332% were attained with the addition of enzymatic fibers, exhibiting lower increments for chemical and mechanical fibers. Enzymatic fibers also showed potential as an alternative to wood and synthetic fibers, while offering environmental and economic advantages.

From the micromechanical analysis of the Young’s modulus, the main following remarks are pointed out: (i) Hirsch and Tsai–Pagano models showed good agreement in the calculus of the fibers’ intrinsic Young’s modulus, reaching values up to 23–24 GPa in enzymatic fibers; (ii) the good performance of enzymatic fibers was ascribed to the presence of lignin in the kappa range of 40 to 50; (iii) composites exhibited a certain degree of anisotropy. Similarities were found between Hirsch and Tsai–Pagano models during the evaluation of the longitudinal and transverse modulus.

## Figures and Tables

**Table 1:** Macro-characteristics of the composites.

Sample	MAPP (wt.%)	Reinforcement (wt.%)	V^f^	E_t_^c^ (GPa)	ε_t_^c^ (%)	ρ^c^ (g/cm^3^)
PP	0	0	0	1.50 ± 0.06	9.28 ± 0.21	0.905
PP ^1^	5	0	0	1.70 ± 0.12	9.91 ± 0.13	0.905
PP ^2^	5	0	0	1.41 ± 0.13	10.62 ± 0.26	0.906
PP + 40% DPF-D	5	40	0.315	3.92 ± 0.04	2.65 ± 0.41	1.033
PP + 60% DPF-D	5	60	0.509	5.45 ± 0.06	2.41 ± 0.29	1.111
PP + 40% DPF-NaOH	5	40	0.307	4.35 ± 0.09	2.73 ± 0.22	1.046
PP + 60% DPF-NaOH	5	60	0.499	6.12 ± 0.10	2.68 ± 0.35	1.134
PP + 40% DPF-E	5	40	0.291	4.57 ± 0.03	3.08 ± 0.29	1.069
PP + 60% DPF-E	5	60	0.480	6.48 ± 0.05	2.91 ± 0.42	1.176
PP + 10% GF	5	10	0.039	2.82 ± 0.08	3.91 ± 0.24	0.966
PP + 20% GF	5	20	0.085	4.21 ± 0.05	3.09 ± 0.37	1.036
PP + 30% GF	5	30	0.137	5.74 ± 0.11	2.84 ± 0.39	1.116
PP + 40% GF	5	40	0.198	6.85 ± 0.09	2.53 ± 0.24	1.210

^1^ Properties of the neat matrix in composites reinforced with 40 wt.% of DPF. ^2^ Properties of the neat matrix in composites reinforced with 60 wt.% of DPF.

**Table 2:** Chemical composition of date palm wastes (DPW) and date palm fibers (DPF).

Sample	Holocellulose (wt.%)	Klason Lignin (wt.%)	Kappa Number	Ashes (wt.%)	Extractives (wt.%)
^1^ DPW-Leaflet [24]	53.01	32.20	-	10.54	4.25
^1^ DPW-Leaf [55]	74.75	15.30	-	1.75	8.20
^1^ DPW-Rachis [24]	66.43	22.53	-	5.96	5.08
^2^ DPF-D	63.5	26.5 ± 0.31	87.5	7.41 ± 0.16	2.59 ± 0.12
^2^ DPF-NaOH	67.7	24.6 ± 0.22	66.6	5.70 ± 0.24	1.98 ± 0.08
^2^ DPF-E	77.4	19.9 ± 0.17	43.9	1.97 ± 0.14	0.72 ± 0.07

^1^ Values reported in the literature. ^2^ Values measured in the present work.

**Table 3:** Intrinsic Young’s modulus of DPF in PP composites and main morphological characteristics.

Material	Reinforcement (wt.%)	l_ww_^f^ (µm)	d^f^ (µm)	E_t_^f^-Hirsch (GPa)	E_t_^f^-TP/HT (GPa)
PP + 40% DPF-D	40	682.0	29.8	16.2	14.1
PP + 60% DPF-D	60	593.0	29.8	17.7	15.3
PP + 40% DPF-NaOH	40	650.1	27.4	20.1	18.4
PP + 60% DPF-NaOH	60	568.5	27.6	21.4	19.5
PP + 40% DPF-E	40	434.5	18.0	23.2	22.4
PP + 60% DPF-E	60	358.2	18.2	24.2	23.7

**Table 4:** Modulus efficiency factor (η), modulus length factor (ηl) and modulus orientation factor (ηo).

Sample	Reinforcement (wt.%)	Hirsch Model			TP/HT Model		
		η	ηl	ηo	η	ηl	ηo
PP + 40% DPF-D	40	0.54	0.93	0.58	0.62	0.93	0.67
PP + 60% DPF-D	60	0.53	0.93	0.57	0.61	0.94	0.65
PP + 40% DPF-NaOH	40	0.51	0.92	0.56	0.56	0.92	0.60
PP + 60% DPF-NaOH	60	0.51	0.93	0.55	0.56	0.93	0.59
PP + 40% DPF-E	40	0.50	0.93	0.53	0.52	0.94	0.55
PP + 60% DPF-E	60	0.50	0.92	0.54	0.51	0.92	0.55
